# Prognostic Factors in Gastrointestinal Leiomyosarcomas: An Analysis Using the Surveillance, Epidemiology, and End Results (SEER) Database

**DOI:** 10.7759/cureus.19447

**Published:** 2021-11-10

**Authors:** Harshavardhan Senapathi, Anthony Morada, Morgan Perry, Ceyda Bertram, Enoch Yeung, Mohammad Sultany, David Bertsch, Burt Cagir

**Affiliations:** 1 Surgery, Guthrie Robert Packer Hospital, Sayre, USA; 2 Surgery, Geisinger Commonwealth School of Medicine, Scranton, USA; 3 Biomedical Research, Guthrie Robert Packer Hospital, Sayre, USA; 4 General Surgery, Guthrie Robert Packer Hospital, Sayre, USA; 5 Surgical Oncology, Guthrie Robert Packer Hospital, Sayre, USA; 6 Colorectal Surgery, Guthrie Robert Packer Hospital, Sayre, USA

**Keywords:** prognostic factors, surgery, survival, leiomyosarcomas, gastrointestinal

## Abstract

Background

Gastrointestinal leiomyosarcomas (LMSs) from intramural smooth muscle are extremely rare, with limited literature. This paper evaluates the epidemiology and survival and prognostic factors in LMSs of the gastrointestinal tract.

Methods

Clinical data from the Surveillance, Epidemiology and End Results (SEER) 18 registry from 2001 to 2016 with additional treatment fields were compared between primary tumor sites using the chi-squared test for categorical variables and ANOVA for continuous variables. A five-year survival rate analysis was performed for overall and cancer-specific survival. Hazard ratios (HRs) were calculated using univariate and multivariate Cox proportional models using the variables age group, tumor location, grade, stage, surgery, and chemotherapy.

Results

We identified a total of 523 patients diagnosed with LMSs of the gastrointestinal tract. The median age of diagnosis was 66 years, with no significant difference between tumor sites for age, sex, and race. The five-year overall survival was 77.3%, and the cancer-specific survival was 90.3%. In the multivariate analysis, grade and stage of tumor were the only factors significantly affecting survival in this cohort.

Conclusion

While surgical status significantly affected survival in the univariate analysis, when adjusted for other factors, the HR for death was not significantly different by surgical therapy. Grade 3 tumors and tumors with distant metastasis at diagnosis were associated with worse survival among these patients.

## Introduction

Leiomyosarcomas (LMSs) make up 10%-20% of all soft tissue tumors and most commonly originate in the uterus, retroperitoneum, and gastrointestinal tract [1]. In 1998, Hirota et al. discriminated leiomyosarcomas (LMSs) from gastrointestinal stromal tumors (GISTs) by describing that true LMSs are negative for CD117 (KIT) and CD 34 and positive for smooth muscle actin or desmin [2]. According to the World Health Organization (WHO) Classification of Tumors, 5th Edition of Digestive System Tumors, only 76 gastrointestinal tract LMSs have been reported since 2000. Within the gastrointestinal tract, LMS tumors frequently occur in the small intestine (40%), colorectum (40%), and more rarely in the stomach (10%) and esophagus (10%), with an incidence equal among males and females [3]. The WHO Classification of Tumors characterizes LMS as aggressive neoplasms with a 40%-80% local recurrence rate, 55%-70% metastasis rate, and 20%-50% mortality rate, depending on the tumor site [3]. Serrano et al. identified LMS grade, location, and stage as crucial independent prognostic factors in survival among these patients [4]. Given the described outcomes, the literature provides little evidence on LMS management due to its rarity. The most common treatment for LMS has been surgical resection with negative margins [5]. LMS and GIST differentiation are essential, as GISTs respond to imatinib, unlike LMS [6].

Based on this information, a broader analysis with a more extensive database could provide more details on the epidemiology and presentation of LMS along with factors that correlate with survival. This paper’s primary goal is to elucidate the epidemiology, survival, and benefit of surgical therapy in patients with primary LMSs in the gastrointestinal tract. We evaluated the overall and cancer-specific survival of LMS using the Surveillance, Epidemiology, and End Results program’s (SEER) 18 registry data from 2001 to 2016 in patients diagnosed with LMS in the gastrointestinal tract using patient demographic data, tumor-specific characteristics, and primary therapy received by the patient.

## Materials and methods

We conducted a retrospective study using data from the SEER Program 18 registry database with additional treatment fields, including chemotherapy information from SEER statistical software [7,]. All patients diagnosed with LMSs originating in the esophagus, stomach, small intestines, colon, or rectum between 2001 and 2016 in the United States were included in the study. Demographic information was collected for age of diagnosis as a continuous variable, along with sex and race as categorical variables. Variables on tumor characteristics were collected for the site of tumor in the gastrointestinal tract, grade of tumor at diagnosis and stage of tumor at diagnosis. The grade of the tumor was categorized based on the level of differentiation described as SEER and scored as 1 for well-differentiated tumors, 2 for moderately differentiated tumors and 3 for poorly differentiated and undifferentiated tumors according to the soft tissue sarcoma scoring system. The stage of the tumor was categorized as localized, regional and distant based on the distance of metastasis as categorized by SEER. Surgical therapy was coded as ‘Yes’ if the patient received any form of intervention endoscopic or invasive and as ‘No’ if the patient received no surgical therapy.

The variables were compared using analysis of variance (ANOVA)/Kruskal Wallis H test for age of diagnosis and chi-squared test statistic for categorical variables between anatomic sites of tumor location. Patients with complete survival data were used to calculate the 5-year cancer-specific survival and overall survival of the patients using Kaplan-Meier survival curves to calculate differences in survival based on surgical therapy. Hazard ratios of variables were calculated using univariate Cox proportional models and multivariate Cox proportional hazard models for adjusted hazard ratios to find independent prognostic factors. All statistical analyses were performed in Rstudio [9].

## Results

There were 523 patients diagnosed with leiomyosarcomas of the digestive tract from 2001 to 2016, and the overall characteristics are shown in Table 1. The median age of patients was 66 years, with equal incidence among males and females. Seventy-eight percent of patients were white, with blacks being the next most common (13.8%), and the rest comprised American Indian, Asian, and Pacific islanders. The majority of the tumors occurred in the small intestines, constituting 31.7%, followed by the stomach, with 28.3%, and the colon, with 26.4%. LMSs were rarely found in the rectum (9.4%) and esophagus (only 5.9%). Forty percent of the tumors were diagnosed in an advanced grade, with another 41% in an unknown grade at diagnosis. A total of 45.9% of tumors were localized, 24.1% were regional, 21.4% had distant metastasis, and the rest were unknown. A total of 82.4% of patients were treated with any kind of surgical therapy. As shown in Table 2, when comparing tumor sites, we found no significant difference by age, sex, or tumor grade at diagnosis. There was a significant difference in the stage of the tumor at presentation, with sarcomas of the stomach and esophagus more commonly found with distant metastasis.

**Table 1:** Overall demographics of patients diagnosed with gastrointestinal leiomyosarcomas from 2001 to 2016 registered on the Surveillance, Epidemiology, and End Results (SEER) Program. *Age is represented as the median (IQR [interquartile range]).

Variable	N (%)
Age*	66.0 (56, 76)
Sex	
Female	258 (49.3)
Male	265 (50.7)
Race	
White	408 (78.0)
Black	72 (13.8)
Other	39 (7.5)
Unknown	4 (0.8)
Site	
Esophagus	22 (4.2)
Stomach	148 (28.3)
Small intestine	166 (31.7)
Colon	138 (26.4)
Rectum	49 (9.4)
Grade	
1	31 (5.9)
2	65 (12.4)
3	212 (40.5)
Unknown	215 (41.1)
Stage	
Localized	240 (45.9)
Regional	126 (24.1)
Distant	112 (21.4)
Unknown	45 (8.6)
Surgery	
No	92 (17.6)
Yes	431 (82.4)

**Table 2:** Comparison of patient demographics by site of the tumor, tumor characteristics and surgical intervention. *Age is represented as the median (IQR), and sex, race, grade, stage and surgery are represented as n (%). p-values for age were calculated by ANOVA and all others using chi-square. statistical tests.

	Esophagus	Stomach	Small intestine	Colon	Rectum	p
Age*:	66.0 (56.25, 78.25)	69.0 (57.75, 80)	64.0 (56, 74)	64.0 (53, 74.75)	65.0 (58, 75)	0.14
Sex						0.073
Female	7 (31.8)	68 (45.9)	77 (46.4)	76 (55.1)	30 (61.2)	
Male	15 (68.2)	80 (54.1)	89 (53.6)	62 (44.9)	19 (38.8)	
Race						0.269
Black	7 (31.8)	20 (13.5)	22 (13.3)	15 (10.9)	8 (16.3)	
White	13 (59.1)	115 (77.7)	133 (80.1)	111 (80.4)	36 (73.5)	
Other	2 (9.1)	13 (8.8)	10 (6.0)	9 (6.5)	5 (10.2)	
Unknown	0 (0.0)	0 (0.0)	1 (0.6)	3 (2.2)	0 (0.0)	
Grade						0.237
1	0 (0.0)	9 (6.1)	11 (6.6)	7 (5.1)	4 (8.2)	
2	3 (13.6)	21 (14.2)	22 (13.3)	13 (9.4)	6 (12.2)	
3	8 (36.4)	44 (29.7)	74 (44.6)	64 (46.4)	22 (44.9)	
Unknown	11 (50.0)	74 (50.0)	59 (35.5)	54 (39.1)	17 (34.7)	
Stage						0.001
Localized	11 (50.0)	68 (45.9)	70 (42.2)	69 (50.0)	22 (44.9)	
Regional	3 (13.6)	17 (11.5)	53 (31.9)	37 (26.8)	16 (32.7)	
Distant	7 (31.8)	43 (29.1)	32 (19.3)	24 (17.4)	6 (12.2)	
Unknown	1 (4.5)	20 (13.5)	11 (6.6)	8 (5.8)	5 (10.2)	
Surgery						<0.001
No	8 (36.4)	52 (35.1)	13 (7.8)	8 (5.8)	11 (22.4)	
Yes	14 (63.6)	96 (64.9)	153 (92.2)	130 (94.2)	38 (77.6)	

**Figure 1:**
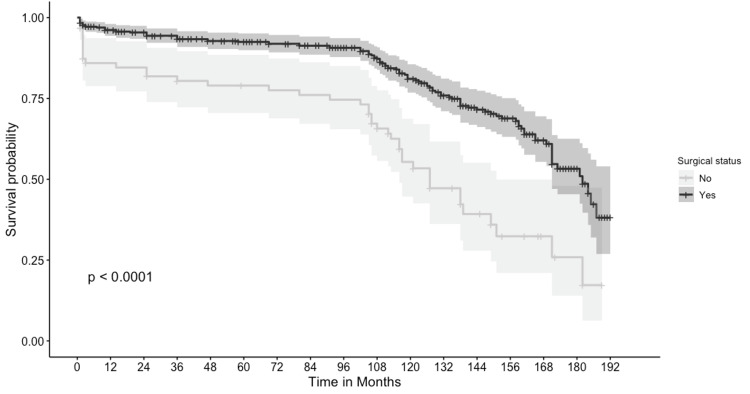
Comparing cancer survival among patients with and without surgery.

**Figure 2:**
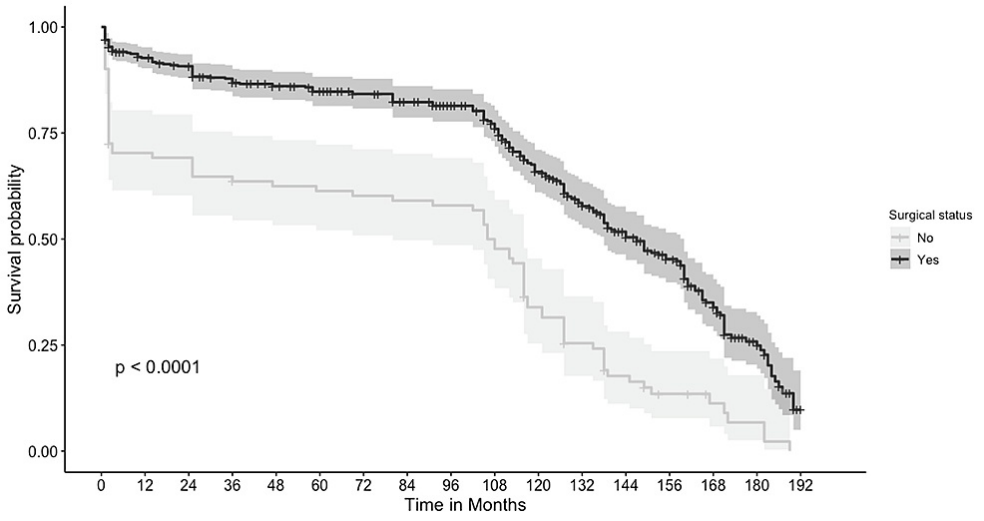
Comparing overall survival among patients with and without surgery.

A total of 521 patients were included in the survival analysis, and two were excluded due to lack of complete survival data. The cancer-specific five-year survival of this cohort was 90.3%, and the five-year overall survival of these patients was 77.3%. Among patients who underwent surgery, the five-year cancer-specific survival and overall survival rates were 92.5% and 84.8%, respectively. Patients who did not have surgery had a five-year overall survival of 61.3% and cancer-specific survival of 79%, which were significantly lower than those of patients who had surgery, with a p-value less than 0.001, as shown in Figure 1 and Figure 2. A total of 297 patients were included in the Cox proportional hazard model after removing patients with unknown grade and stage. When hazard ratios were calculated using the above variables, tumor grade, tumor stage, and surgical status significantly affected survival, as shown in Table 3. In the multivariate analysis, only the stage and grade of tumors were the only independent factors affecting survival, with hazard ratios shown in Table 3. Grade 3 sarcomas had 7.71 (95% CI: 1.06, 56.02) times the hazard of death compared to Grade 1 sarcomas. Sarcomas with distant metastasis had 3.59 times the hazard of death compared to patients with localized tumors, with a 95% CI of 1.89 and 6.84.

**Table 3:** Univariate and multivariate hazard ratios with 95% confidence intervals and p-values. *Significantly affected hazard ratio for death in univariate analysis. **Significantly affected hazard ratio in the multivariate analysis.

	Univariate hazard ratios	Multivariate hazard ratios
Age	1.00 (0.99-1.02, p=0.821)	1.01 (0.99-1.02, p=0.450)
Sex		
Male	-	-
Female	0.97 (0.63-1.50, p=0.902)	1.24 (0.78-1.96, p=0.357)
Site		
Small intestine	-	-
Colon	0.78 (0.44-1.40, p=0.404)	0.61 (0.33-1.12, p=0.113)
Esophagus	1.17 (0.41-3.35, p=0.765)	0.92 (0.32-2.66, p=0.881)
Rectum	0.69 (0.30-1.59, p=0.385)	0.58 (0.25-1.38, p=0.220)
Stomach	1.41 (0.82-2.44, P=0.214)	1.31 (0.72-2.40, p=0.374)
Grade		
1	-	-
2	4.02 (0.51-31.43, p=0.185)	3.92 (0.50-30.75, p=0.193)
3	9.64 (1.34-69.41, p=0.024)*	7.71 (1.06-56.02, p=0.043)**
Stage		
Localized	-	-
Regional	2.54 (1.43-4.50, p=0.001)*	2.57 (1.42-4.66, p=0.002)**
Distant	4.66 (2.66-8.16, p<0.001)*	3.59 (1.89-6.84, p<0.001)**
Surgery		
No	-	-
Yes	0.23 (0.12-0.42, p<0.001)*	0.61 (0.28-1.33, p=0.212)

## Discussion

This study describes the fundamental characteristics of patients with intestinal LMS. We included patients diagnosed with gastrointestinal LMSs from 2001 due to discrepancies in the classification of these tumors before that time. When faceted by the site of the digestive tract, LMS occupied the stomach, small bowel and colon and was found very rarely in the esophagus and rectum. There was no significant difference in age, sex, or race of patients with gastrointestinal LMSs between all tumor sites. Patients who underwent surgery had significantly better cancer-specific survival and overall survival than patients who did not undergo surgery, as shown in the survival curves (Figures 1, 2) and univariate Cox proportional model (Table 3). In the univariate Cox proportional hazard model, we observed grade, stage, chemotherapy, and surgical therapy to significantly affect survival. In the multivariate analysis, tumor grade and tumor stage were the only independent prognostic factors affecting survival in these patients.

There is currently limited knowledge on the epidemiology, diagnosis, and treatment of LMS in the intestines. Physicians generally suspect LMS when the tumor disrupts normal tissue planes, have cystic spaces with enlarged lymph nodes and contain a varying degree of necrosis, calcification, and heterogeneous contrast enhancement [6]. In Miettinen’s case studies on patients diagnosed with gastrointestinal LMSs, the median age at diagnosis for esophageal LMSs was 63 years, small intestines was 55, colon was 61, and rectum was 58 years [1,10-12]. We did not find any large studies focusing on gastric LMS. In comparison, our median (interquartile range) age at diagnosis was 66 (18) years, with a significant difference among all five sites. Regarding the presentation of disease at diagnosis, 40.5% of these sarcomas being diagnosed with grade 3 in these patients exemplify the late recognition of these cancers. However, there were no significant differences in the grade at diagnosis based on the site of the sarcoma.

After recognizing a suspected LMS neoplasm, surgery is the primary treatment of choice. Most providers recommend obtaining negative margins, given the high recurrence rates of these tumors [5]. In our univariate analysis, patients with surgical therapy had a significantly lower hazard of death than patients without surgery; however, in our multivariate analysis, there was no difference in the risk of death when adjusted for other factors. Based on the findings of this cohort, grade and stage at the time of diagnosis seem to have a crucial role in the prognosis of the patients. The efficacy of chemotherapy and radiotherapy is unclear and hence was not included in our analysis [5]. The overall survival calculated on 29 cases of GI tract LMSs by Yamamoto et al. showed an overall survival of 51.6% at five years after surgery, which is much lower than our cohort with an overall survival of 84.8% and cancer-specific survival of 90.7% [13]. The survival results of Yamamoto could be due to the more advanced stages and recurrent cases and hence the lower survival.

This analysis is the first large-scale analysis on the epidemiology and cancer-specific survival of these sarcomas using National Cancer Institute data. We believe that the patient data are accurate and assume it represents the United States Gastrointestinal LMS population. As a retrospective study using a curated national database, our study design carries inherent limitations. While the SEER registry collects cancer incidence data at a national level, its generalizability to describe all cancer types restricts potentially important variables for specific tumor types. For example, the current literature on gastrointestinal LMS has suggested that variables such as tumor size, mitotic count, and adjuvant therapies could be associated with overall survival. Yamamoto et al. showed that patients with tumors greater than 5 cm had less overall survival than patients with tumors less than 5 cm and showed no difference in survival based on tumor size above and below 10 cm or by mitotic counts [13]. However, we could not obtain these variables in SEER because of the lack of data or the large volume of missing data. Next, we could not categorize surgery based on the level of invasion due to the low specificity of SEER surgical codes performed on patients; hence, we decided to make it a binary variable. The exclusion of these variables confirmed in the LMS literature might affect our multivariate analysis due to its influence by confounding variables.

## Conclusions

This study is the first large-scale study analyzing the survival of LMS in the gastrointestinal tract. The median age at diagnosis is between the fifth and sixth decades of life, and the incidence is equal between males and females. A high proportion of patients were diagnosed at an advanced stage of disease due to the late onset of symptoms. A higher tumor grade and tumor stage independently affected the prognosis of patients. Surgical therapy significantly affected patient survival in the univariate analysis but did not affect survival independently in the multivariate analysis. A larger study with more variables would give a better picture of the importance of surgery for patients when adjusted for other factors. We believe there is a wealth of information unexplored in gastrointestinal leiomyosarcomas, and there is a need for more information to be collected for a better understanding of the disease in terms of assessing the severity and benefits of treatment.

## References

[1] Miettinen M, Sarlomo-Rikala M, Sobin LH, Lasota J (2000). Gastrointestinal stromal tumors and leiomyosarcomas in the colon: a clinicopathologic, immunohistochemical, and molecular genetic study of 44 cases. Am J Surg Pathol.

[2] Hirota S, Isozaki K, Moriyama Y (1998). Gain-of-function mutations of c-kit in human gastrointestinal stromal tumors. Science.

[3] Klimstra D, Klöppel G, La Rosa S, Rindi G (2019). Classification of neuroendocrine neoplasms of the digestive system. WHO Classification of tumours, 5th Edition.

[4] Serrano C, George S (2013). Leiomyosarcoma. Hematol Oncol Clin North Am.

[5] Fairweather M, Raut CP (2015). Surgical management of GIST and intra-abdominal visceral leiomyosarcomas. J Surg Oncol.

[6] Hilal L, Barada K, Mukherji D, Temraz S, Shamseddine A (2016). Gastrointestinal (GI) leiomyosarcoma (LMS) case series and review on diagnosis, management, and prognosis. Med Oncol.

[7] (2021). SEER*Stat Software. http://seer.cancer.gov/seerstat/.

[8] (2020). SEER. Surveillance, Epidemiology, and End Results (SEER) Program (www.seer.cancer.gov) SEER*Stat Database: Incidence - SEER Research Data, 9 Registries, Nov 2019 Sub (1975-2017) - Linked To County Attributes - Time Dependent (1990-2017) Income/Rurality, 1969-20. SEER Research Data.

[9] Core R Team (2019). Core R Team. A Language and Environment for Statistical Computing. R Found Stat Comput.

[10] Miettinen M, Furlong M, Sarlomo-Rikala M, Burke A, Sobin LH, Lasota J (2001). Gastrointestinal stromal tumors, intramural leiomyomas, and leiomyosarcomas in the rectum and anus: a clinicopathologic, immunohistochemical, and molecular genetic study of 144 cases. Am J Surg Pathol.

[11] Miettinen M, Kopczynski J, Makhlouf HR (2003). Gastrointestinal stromal tumors, intramural leiomyomas, and leiomyosarcomas in the duodenum: a clinicopathologic, immunohistochemical, and molecular genetic study of 167 cases. Am J Surg Pathol.

[12] Miettinen M, Sarlomo-Rikala M, Sobin LH, Lasota J (2000). Esophageal stromal tumors: a clinicopathologic, immunohistochemical, and molecular genetic study of 17 cases and comparison with esophageal leiomyomas and leiomyosarcomas. Am J Surg Pathol.

[13] Yamamoto H, Handa M, Tobo T (2013). Clinicopathological features of primary leiomyosarcoma of the gastrointestinal tract following recognition of gastrointestinal stromal tumours. Histopathology.

